# Retrieval of High Added Value Natural Bioactive Coumarins from Mandarin Juice-Making Industrial Byproduct

**DOI:** 10.3390/molecules26247527

**Published:** 2021-12-12

**Authors:** Eleni D. Myrtsi, Apostolis Angelis, Sofia D. Koulocheri, Sofia Mitakou, Serkos A. Haroutounian

**Affiliations:** 1 Laboratory of Nutritional Physiology and Feeding, Department of Animal Science, School of Animal Biosciences, Agricultural University of Athens, Iera Odos 75, 11855 Athens, Greece; elenamirtsi@aua.gr (E.D.M.); skoul@aua.gr (S.D.K.); 2 Division of Pharmacognosy and Chemistry of Natural Products, Faculty of Pharmacy, University of Athens, Panepistimiopolis Zografou, 15771 Athens, Greece; aangjel@pharm.uoa.gr (A.A.); mitakou@pharm.uoa.gr (S.M.)

**Keywords:** mandarin (*Citrus reticulata*), circular economy, coumarin, furanocoumarin, heraclenol, auraptene, bergamottin, 8-geranylpsoralen

## Abstract

Cold pressed essential oil (CPEO) of mandarin (*Citrus reticulata* Blanco), a by-product of the juice-making industrial process known to contain large amounts of polymethoxyflavones, was exploited for its content in high added value natural coumarins. The study herein afforded a method referring to the evaporation of CPEO volatile fraction under mild conditions (reduced pressure and temperature below 35 °C) as azeotrope with isopropanol. This allowed the isolation of high added value coumarins from the non-volatile fragment using preparative High Performance Liquid Chromatography (HPLC). Pilot-scale application of this procedure afforded for each kg of CPEO processed the following natural bioactive coumarins in chemically pure forms: heraclenol (38–55 mg), 8-gerayloxypsoralen (35–51 mg), auraptene (22–33 mg), and bergamottin (14–19 mg). The structures of coumarins were verified by Nuclear Magnetic Resonance (NMR) spectroscopy and HPLC co-injection with authentic standards. Thus, the low market value mandarin CPEO with current value of 17 to 22 EUR/kg can be valorized through the production of four highly bioactive natural compounds worth 3479 to 5057 EUR/kg, indicating the great potentials of this methodology in the terms of the circular economy.

## 1. Introduction

Coumarins belong to plant-derived secondary metabolites that structurally are classified into benzopyrone derivatives. They are known as molecules exhibiting a broad spectrum of bioactivities, including anticancer [1], anti-HIV [2], antioxidant [3,4,5,6], anticoagulant [7], antiviral [8,9,10], antimicrobial [11,12], anti-inflammatory [13] and Central Nervous System stimulating properties [14]. Additionally, various coumarins are being used as odorants by the cosmetic and food industries [15,16]. These bioactivities are frequently combined with crucial features such as high bioavailability, broad spectrum of activities, low toxicity, and lack of drug resistance development [17], pointing to coumarins as intriguing leads for the development of pharmaceuticals for the treatment of numerous health disorders and diseases. Consequently, vigorous research activity has been initiated towards the discovery of bioactive molecules containing the coumarin structural backbone either from natural resources [18] and/or synthetic pathways [19,20,21]. In this respect, many medicinal and aromatic-edible plants belonging to widely distributed plant families, such as Rutaceae, Moraceae, Apiaceae, and Fabaceae, have been determined as plant sources for the isolation of natural coumarins [22,23,24,25]. Most of them are included in the catalog of well-known elements of the herbal medicine repertories in Europe, Asia, and America, displaying established ethnomedicinal applications in traditional medicine systems, such as Ayurveda Medicine and Traditional Chinese Medicine [24]. On the other hand, during the last decades, an intense research effort has been devoted to the development of synthetic pathways for the efficient approach of coumarin derivatives. Consequently, a broad diversity of novel synthetic derivatives and analogs of coumarins has been prepared [13,20,26,27] providing leads for utilization in photochemotherapy, in antitumor and anti-HIV therapies, as anti-coagulant, anti-inflammatory, and anti-bacterial agents and as CNS stimulants [1,2,3,8,9,20,28,29].

The molecule of natural coumarin (chemical name 2H-chromene-2-one or 1,2-benzopyrone) was firstly isolated in 1820 from the Tonka bean seeds of *Dipteryx* (*Coumarouna*) *odorata*, Fabaceae/Leguminosae family. It was named from the French term *coumarou*, which is used for naming the plant. The first synthesis of coumarin was accomplished in 1868, initiating a campaign for its utilization by pharmaceutical industries as a precursor for the synthesis of various synthetic anticoagulants [24]. Ever since, the number of in-use natural and synthetic coumarins has grown enormously, leading to the division of their diversity into the following subclasses [14]: simple coumarins (e.g., coumarin and limettin), linear furanocoumarins (e.g., imperatorin and isopimpinellin), angular furanocoumarins (e.g., angelicin), linear pyranocoumarins (e.g., xanthyletin) and angular pyranocoumarins (e.g., seselin).

Furanocoumarins comprise the larger family of coumarin derivatives, which are present in higher plants. Their structural skeleton is characterized by a furan ring, attached on carbons C-6 and C-7 (linear type) or C-7 and C-8 (angular type) of the coumarin backbone [30]. The first studied furanocoumarin is 5-methoxypsoralen, which was isolated in 1838 from bergamot oil. Furanocoumarins have been used in folk medicine for a long time. For example, the treatment of leukoderma (vitiligo) is achieved by Indians using poultices from the furanocoumarin rich plant *Psoralea corylifolia* as described in their sacred book Atharva Veda and by ancient Egyptians from *Ammi majus* according to their writings [31]. Furanocoumarins also act as strong photosensitizers in humans, causing severe phytophotodermatitis incidences after skin contact and/or ingestion, followed by exposure to sun-derived UV-A radiation [23,32]. Under these conditions, they react with nucleic bases of DNA causing most of the plant-derived phototoxic reactions of humans [33]. Therefore, furanocoumarins are frequently used as potent photochemotherpeutic agents [34,35]. Finally, furanocoumarins are known to possess various additional health beneficial effects, such as antioxidant [3,4,5], anticancer [1,36], antiviral [8,9,10] activities, and insecticidal ability [37,38].

*Citrus* crops are included among the richest sources of natural compounds, including coumarins and furanocoumarins [39]. Their global annual production exceeds 150 Mt, of which a significant percentage (almost 40%) is used for the production of juice-based products [40]. This endeavor generates a considerably large volume of byproducts since only 50% of the fresh fruit mass is transformed into juice [41]. The remaining is currently of low economic value, although its content of soluble sugars, cellulose, hemicellulose, pectin, flavonoids, and essential oils [42] is indicative of the great potential for utilization as raw materials of biorefineries [43]. To date, the cold-pressed essential oil (CPEO) constitutes the only valorized byproduct of the *Citruses* juice-making process, since it is widely used as a flavoring and fragrance agent by food, beverage, cosmetic, and pharmaceutical industries due to its characteristic aroma profile [44,45]. Its current market value ranges from 7.5 to 42 EUR/kg [46], depending on the processed fruit and the content of its volatile fraction. The latter represents the larger and most studied fraction of *Citruses* CPEOs, while the non-volatile residue, consisting of sterols, fatty acids, waxes, carotenoids, coumarins, psoralens, and flavonoids [47], remains unexploited. A recent review of 2021 [48] collected all relevant literature data concerning the exploitation of *Citruses* CPEOs as the source of bioactive psoralens, coumarins, and polymethoxyflavones (PMFs). The respective data revealed the presence of these compounds in the essential oils of oranges, bergamot, lemons, and grapefruits. On the contrary, the literature reports for mandarin (*Citrus reticulata*) CPEO, a well-known agricultural commodity, reveal only the presence of PMFs. The latter belong to an important class of bioactive molecules that are isolated in large amounts from the peel of various citrus fruits (sweet and bitter orange, grapefruit, mandarin), making economically unprofitable their recovery from the essential oil of mandarin.

The aim of this study is to exploit, for the first time, the mandarin CPEO as a source for the efficient retrieval of bioactive coumarins and furanocoumarins. It must be noted that the presence of these bioactive molecules in mandarin fruits is well known but there are no reports concerning their presence in the essential oil of mandarin [48,49,50]. This endeavor is of high economic interest since, according to the Food and Agricultural Organization (FAO) data [40], it is estimated that annually 30.7 Mt of mandarin is processed by the food industry for the production of mandarin juice-based products. This procedure generates as a byproduct the respective CPEO, in yields ranging from 0.4 to 0.6 mL per kg of fruit [46]. Thus, the study of mandarin CPEO in order to determine its content in high added value bioactive natural coumarins and furanocoumarins and the possible development of an efficient method for their facile retrieval is of significant economic interest.

## 2. Results

### 2.1. Isolation of Bioactive Molecules from Mandarin CPEO

**Figure 1:**
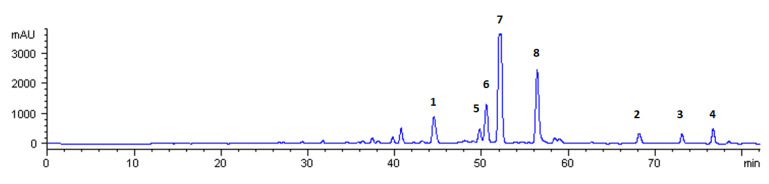
HPLC preparative chromatogram of the isolated natural compounds. Peaks correspond to heraclenol (**1**, 40.7 min), 8-geranyloxypsoralene (**2**, 68.1 min), auraptene (**3**, 72.9 min), bergamottin (**4**, 76.5 min), nobiletin (**5**, 50.2 min), 5,6,7,4′-tetramethoxyflavone (**6**, 50.7 min), 3,5,6,7,8,3′,4′-heptamethoxyflavone (**7**, 51.9 min), and tangeretin (**8**, 56,3 min).

**Figure 2:**
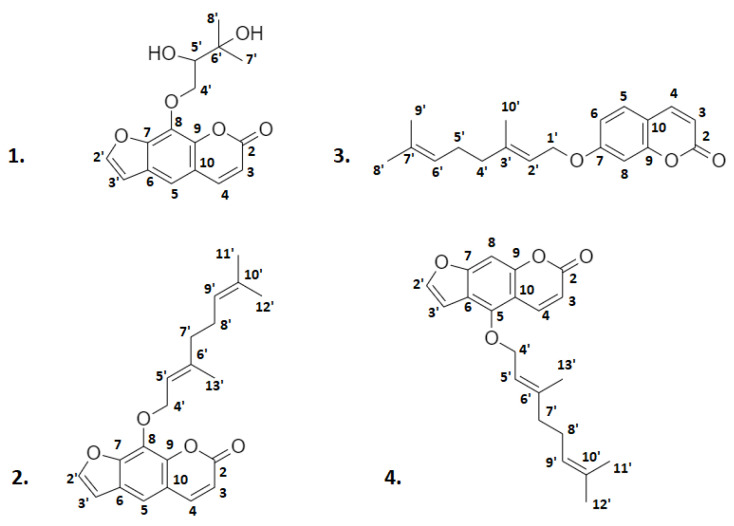
Structures of retrieved natural coumarins and furanocoumarins. **1**: heraclenol; **2**: 8-geranyloxypsoralene; **3**: auraptene; **4**: bergamottin.

**Figure 3:**
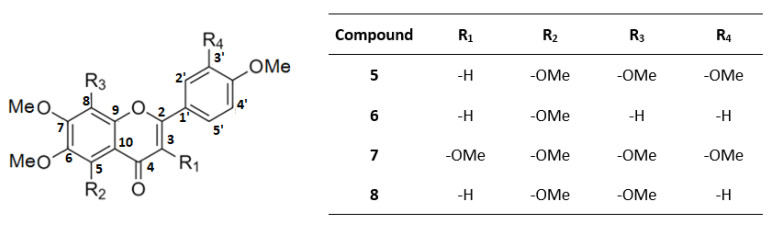
Chemical structures of PMFs isolated from mandarin CPEO. **5**: nobiletin, **6**: 5,6,7,4′-tetramethoxyflavone, **7**: 3,5,6,7,8,3′4′-heptamethoxyflavone, and **8**: tangeretin.

The volatile fraction of mandarin CPEO, consisting mainly of d-limonene [45], was removed herein by evaporation under reduced pressure at very low temperature (below 35 °C) in the form of an azeotropic mixture with isopropanol. In this way, we were able to avoid the utilization of the classic hydro–distillation procedure, which is commonly applied for the separation of volatile essential oils and requires heating at 100 °C. Thus, it was feasible to obtain from 1 kg of CPEO approximately 50 g of the non-volatile fraction containing—intact—the majority of the contained sensitive bioactive molecules. Then, the mixture was kept in a freezer for 24 h in order to precipitate the majority of the unwanted organic material, which is comprised of fatty acids, waxes, sugars, cellulose, and hemicellulose. The supernatant was separated by decantation, dissolved in methanol, and introduced into preparative high performance liquid chromatography (HPLC) to afford the following eight bioactive molecules (Figure 1) that were separated into the following two distinct classes of compounds as amorphous colorless solids: *Coumarins* and *furanocoumarins* (Figure 2), eluted as heraclenol (**1**), 8-geranyloxypsoralen which is also known also as xanthotoxol geranyl ether (**2**), auraptene (**3**) and bergamottin (**4**); *Polymethoxyflavones* (PMFs, (Figure 3)), eluted as nobiletin (**5**), 5,6,7,4′-tetramethoxyflavone (**6**), 3,5,6,7,8,3′,4′-heptamethoxyflavone (**7**), and tangeretin (**8**).

The efficacy of the method developed was also tested and verified in pilot scale experiments using a 25 L capacity rotary evaporator for the separation of the non-volatiles fraction of 4 kg of mandarin CPEO and the calculation of the retrieval yields for each compound.

### 2.2. Identification of the Isolated Compounds

The chemical structures of the retrieved molecules were elucidated by NMR spectroscopy and verified through HPLC co-injection with solutions containing the standard authentic compound. Specifically, for heraclenol, the ^1^H–NMR spectra revealed the existence of two methyl groups attached on carbon 6′ as singlets at 1.29 and 1.35 ppm, the protons on carbons 4′ and 5′ (4.66 and 3.30, respectively) and the furanocoumarin skeletal protons that resonate in the area 6.00–8.50 ppm. These assignments were based on the respective correlated spectroscopy (COSY) spectrum which verified the connections between vicinal carbons as signals between protons H-3 and H-4, H-2′ and H-3′, H-4′ and H-5. Finally, the existence of carbons 3, 4, 5, 2′, 3′, 4′, 5′, 7′, and 8′ was determined with the heteronuclear multiple-quantum coherence (HMQC) spectrum, while the remaining carbons were located with the aid of heteronuclear multiple bond correlation (HMBC) spectrum.

In the ^1^H–NMR spectra of 8-geranyloxypsoralene, the two methyl groups attached on carbon 10′ resonate as singlets at 1.57 and 1.63 ppm, while the methyl group on carbon 6′ appears as a singlet at 1,66 ppm. The double bond protons (H-5′ and H-9′), along with methylene protons on C-4′ that are attached to the oxygen atom were detected at chemical shifts between 5–5.60 ppm, while the protons of furanocoumarin skeleton appear in the area 6–8.20 ppm. On the other hand, the COSY spectrum revealed the connections between protons H-4′ and H-5′, H-7′ and H-8′, H-8′ and H-9′, all indicative of their vicinity and the signals between H-3 and H-4, H-2′ and H-3′ that are characteristic of the furanocoumarin carbon skeleton. The HMQC spectra contributed to the determination of carbons bearing protons and indicated that H-4′, H-5′, H-9′, H-7′, and H-8′ although displaying similar chemical shift values in ^1^H–NMR, were attached to different carbons. The remaining carbon atoms of the molecule were determined with the HMBC spectrum.

The protons of the three methyl groups of auraptene, attached to carbons 8′, 9′, and 10′ appear in ^1^H–NMR spectra as singlets at 1.62, 1.66, and 1.77 ppm respectively, while the methylene protons attached to carbons 4′, 5′ resonate as multiplets at 2.10 and 2.13 ppm. The double bond protons on C-2′ and C-6′ are recorded as triplets at 5.48 and 5.11 ppm, while the methylene protons H-1′ linked to carbon attached to oxygen appear as a broad doublet at 4.66 ppm. Finally, coumarin protons resonate in the area between 6.20–7.80 ppm. The COSY spectrum verified the vicinal interactions between H-1′, H-2′, and H-4′, H-6′ as well as those on coumarin rings as H (6.90 ppm), H (7.51 ppm), and H (6.21 ppm), H (7.80 ppm). The HMQC spectrum was used for the determination of carbons bonded directly with protons in order to reveal the methylene protons and HMBC was used for the designation of the remaining carbons of the molecule.

For the bergamottin molecule, the ^1^H–NMR spectrum revealed three methyl groups, two attached on carbons 10′ (1.60 and 1.66 ppm) and 6′ (1.68 ppm) and methylene protons attached to carbons 7′ and 8′ appear as multiplets at 2.10 and 2.08 ppm. The double bond protons H-5′ and H-9′ attached to double bonds and resonated as triplets at 5.55 and 5.07, respectively, while H-4′ protons on a carbon vicinal to oxygen atom appeared as a doublet at 5.00 ppm. All furanocoumarin protons were detected at 6.20–8.20 ppm. The COSY spectrum is indicative of vicinal interactions between H-5′, H-9′, and H-8′, H-5′, along with those of furanocoumarin carbon skeleton as H (6.25 ppm), H (8.18 ppm), and H (7.13 ppm), H (7.75 ppm). The HMQC spectrum contributed to the determination of carbons bonded directly with protons and especially the designation of methylene protons, while HMBC was used for the designation of the remaining carbons of the molecule.

**Heraclenol** (**1**)**:** ^1^H–NMR (CD_3_CN, 600 MHz) δ: 8.27 (d, H-4, *J* = 9.9 Hz), 7.77 (d, H-3′, *J* = 2.4 Hz), 7.26 (s, H-5), 7.14 (d, H-2′, *J* = 2.4 Hz), 6.30 (d, H-3, *J* = 9.9 Hz), 4.66 (m, H-4′), 3.30 (m, H-5′), 1.35 (s, H-7′), 1.29 (s, H-8′).^13^C–NMR (CD_3_CN, 150 MHz) δ: 161.6 (C-2), 158.6 (C-7), 153.3 (C-9), 149.4 (C-8), 146.4 (C-3′), 139.9 (C-4), 115.0 (C-6), 113.5 (C-3), 108.1 (C-10), 105.5 (C-2′), 94.9 (C-5), 73.4 (C-4′), 61.6 (C-5′), 58.5 (C-6′), 24.2 (C-7′), 19.0 (C-8′).

**8-Geranyloxypsoralen** (**2**)**:** ^1^H–NMR (CD_3_CN, 600 MHz) δ: 7.95 (d, H-4, *J* = 9.6 Hz), 7.84 (d, H-2′, *J* = 2.2 Hz), 7.55 (s, H-5), 6.96 (d, H-3′, *J* = 2.2 Hz), 6.35 (d, H-3, *J* = 9.6 Hz), 5.55 (t, H-5′, *J* = 7.2 Hz), 5.00 (d, H-4′, *J* = 9.6 Hz), 5.00 (d, H-9′, *J* = 9.6 Hz), 2.04 (m, H-7′), 2.04 (m, H-8′), 1.66 (s, H-13′), 1.63 (s, H-12′), 1.57 (s, H-11′). ^13^C–NMR (CD_3_CN, 150 MHz) δ: 161.0 (C-2), 149.1 (C-7), 147.7 (C-2′), 145.0 (C-4), 144.4 (C-9), 143.0 (C-6′), 132.3 (C-10′), 131.4 (C-8), 126.6 (C-6), 124.4 (C-9′), 120.2 (C-5′), 117.4 (C-10), 114.9 (C-3), 114.7 (C-5), 107.5 (C-3′), 70.4 (C-4′), 34.8 (C-7′), 26.9 (C-8′), 25.4 (C-12′), 17.4 (C-11′), 16.4 (C-13′).

**Auraptene** (**3**)**:** ^1^H–NMR (CD_3_CN, 600 MHz) δ: 7.80 (d, H-4, *J* = 9.5 Hz), 7.51 (d, H-5, *J* = 8.9 Hz), 6.90 (dd, H-6, *J* = 8.9, 2.3 Hz), 6.89 (d, H-8, *J* = 2.3 Hz), 6.21 (d, H-3, *J* = 9.5 Hz), 5.48 (t, H-2′, *J* = 6.6 Hz), 5.11 (t, H-6′, *J* = 6.8 Hz), 4.66 (brd, H-1′, *J* = 6.5 Hz), 2.13 (m, H-5′), 2.10 (m, H-4′), 1.77 (s, H-10′), 1.66 (s, H-9′), 1.62 (s, H-8′). ^13^C–NMR (CD_3_CN, 150 MHz) δ: 163.1 (C-7), 161.6 (C-2), 156.9 (C-9), 144.2 (C-4), 143.0 (C-3′), 132.7 (C-7′), 129.5 (C-5), 124.2 (C-6′), 119.4 (C-2′), 113.8 (C-10), 113.1 (C-6), 112.9 (C-3), 101.9 (C-8), 65.8 (C-1′), 39.6 (C-4′), 26.4 (C-5′), 25.2 (C-9′), 17.2 (C-8′), 16.3 (C-10′).

**Bergamottin** (**4**)**:** ^1^H–NMR (CD_3_CN, 600 MHz) δ: 8.18 (d, H-4, *J* = 9.8 Hz), 7.75 (d, H-3′, *J* = 2.3 Hz), 7.20 (s, H-8), 7.13 (d, H-2′, *J* = 2.3 Hz), 6.25 (d, H-3, *J* = 9.8 Hz), 5.55 (t, H-5′, *J* = 7.1 Hz), 5.07 (t, H-9′, *J* = 6.8 Hz), 5.00 (d, H-4′, *J* = 6.9 Hz), 2.10 (m, H-7′), 2.08 (m, H-8′), 1.68 (s, H-13′), 1.66 (s, H-11′), 1.60 (s, H-12′). ^13^C–NMR (CD_3_CN, 150 MHz) δ: 161.4 (C-2), 158.6 (C-7), 153.7 (C-9), 150.1 (C-5), 146.2 (C-3′),143.9 (C-6′), 140.7 (C-4), 133.2 (C-10′), 125.0 (C-9′), 120.4 (C-5′), 115.5 (C-6), 113.8 (C-3), 106.1 (C-2′), 108.6 (C-10), 94.7 (C-8), 70.8 (C-4′), 40.3 (C-7′), 27.2 (C-8′), 25.8 (C-11′), 17.9 (C-12′), 17.0 (C-13′).

Accordingly, the respective NMR data for the isolated of PMFs shown in Figure 3, are:

**Nobiletin** (**5**)**:** ^1^H–NMR (CD_3_CN, 600 MHz) δ: 7.62 (dd, H-6′, *J* = 8.5/2.2 Hz), 7.49 (d, H-2′, *J* = 8.2 Hz), 7.09 (d, H-5′, *J* = 8.5 Hz), 6,62 (s, H-3), 4.05 (s, 7-OMe), 4.02 (s, 5-OMe), 3.93 (s, 3′-OMe), 3.91 (s, 4′-OMe), 3.91 (s, 6-OMe), 3.85 (s, 8-OMe). ^13^C–NMR (CD_3_CN, 150 MHz) δ: 177.0 (C-4), 161.6 (C-2), 152.9 (C-6), 152.2 (C-7), 150.2 (C-3′), 148.7 (C-8), 147.5 (C-9), 144.6 (C-4′), 138.9 (C-5), 124.7 (C-1′), 119.5 (C-6′), 115.4 (C-10), 111.8 (C-5′), 109.1 (C-2′), 106.4 (C-3), 61.6 (5-OMe), 61.6 (8-OMe), 61.4 (6-OMe), 61.2 (7-OMe), 55.6 (3′-OMe), 55.6 (4′-OMe).

**5, 6, 7, 4′-Tetramethoxyflavone** (**6**)**:** ^1^H–NMR (CD_3_CN, 600 MHz) δ: 7.95 (dd, H-2′, *J* = 6.4/2.2 Hz), 7.95 (dd, H-6′, *J* = 6.4/2.2 Hz), 7.08 (dd, H-3′, *J* = 6.4/2.2 Hz), 7.08 (dd, H-5′, *J* = 6.4/2.2 Hz), 7.03 (s, H-8), 6,54 (s, H-3), 3.97 (s, 7-OMe), 3.89 (s, 4′-OMe) 3.88 (s, 5-OMe), 3.85 (s, 6-OMe). ^13^C NMR (CD_3_CN, 150 MHz) δ: 176.9 (C-4), 162.8 (C-4′), 161.7 (C-2), 158.6 (C-7), 155.2 (C-9), 152.8 (C-5), 144.4 (C-5′), 144.4 (C-3′), 140.9 (C-6),127.7 (C-6′), 127.7 (C-2′), 124.4 (C-1′), 113.1 (C-10), 106.4 (C-3), 96.9 (C-8), 61.5 (5-OMe), 61.5 (6-OMe), 56.1 (7-OMe), 55.2 (4′-OMe).

**3, 5, 6, 7, 8, 3′,4′-Heptamethoxyflavone** (**7**)**:** ^1^H–NMR (CD_3_CN, 600 MHz) δ: 7.95 (dd, H-6′, *J* = 6.4/2.2 Hz) 7.53 (s, H-2′), 6.89 (d, H-5′, *J* = 7.3 Hz), 3.84 (s, 7-OMe) 3.65 (s, 3-OMe), 3.76 (s, 5-OMe), 3.69 (s, 3′-OMe), 3.67 (s, 6-OMe), 3.67 (s, 4′-OMe), 3.63 (s, 8-OMe). ^13^C–NMR (CD_3_CN, 150 MHz) δ: 172.3 (C-4), 151.5 (C-2), 151.4 (C-7), 151.3 (C-3′), 149.9 (C-4′), 147.9 (C-9), 147.8 (C-3), 143.8 (C-6), 140.6 (C-8), 137.9 (C-5), 123.5 (C-1′), 121.7 (C-6′), 114.4 (C-10), 111.5 (C-5′), 111.1 (C-2′), 61.5 (5-OMe), 61.2 (6-OMe), 61.1 (7-OMe), 59.0 (8-OMe), 55.3 (3′-OMe), 53.5 (4′-OMe).

**Tangeretin** (**8**)**:** ^1^H–NMR (CD_3_CN, 600 MHz) δ: 7.95 (s, H-2′), 7.95 (s, H-6′), 7.13 (s, H-5′), 7.09 (s, H-3′), 6,56 (s, H-3), 4.08 (s, 7-OMe), 4.01 (s, 5-OMe), 3.91 (s, 6-OMe), 3.89 (s, 4′-OMe), 3.85 (s, 8-OMe). ^13^C–NMR (CD_3_CN, 150 MHz) δ: 177.1 (C-4), 163.0 (C-4′), 161.8 (C-2), 152.3 (C-7), 148.7 (C-8), 147.7 (C-9), 144.8 (C-6), 139.2 (C-5), 128.3 (C-2′), 128.3 (C-6′), 124.5 (C-1′), 115.9 (C-3′), 115.9 (C-5′), 115.6 (C-10), 107.2 (C-3), 62.3 (5-OMe), 62.2 (8-OMe), 61.9 (6-OMe), 61.6 (7-OMe), 55.9 (4′-OMe).

## 3. Discussion

The retrieval of natural products from agro-industrial byproducts constitutes one of the cornerstones of the circular economy. The application of different isolation techniques allows the retrieval of a large variety of bioactive molecules from complex mixtures. Although the extraction and isolation of coumarins from CPEOs of *citrus* fruits, a byproduct of the juice-making process, has been known for a longtime [23,47,48], the CPEO of mandarin (*Citrus reticulata*) remains somehow less studied. For this byproduct, only the presence of PMFs has been reported until today [48], while there are no studies and reports for the presence of other bioactive molecules with an established presence in mandarin fruit, such as coumarins and furanocoumarins. The method developed herein has the advantage that the evaporation of CPEO’s volatile fragment is performed at low temperatures, preventing the decomposition of thermally sensitive molecules. Thus, the application of this method in this agro-industrial byproduct provided—except for the PMF molecules of nobiletin (**5**), 5, 6, 7, 4′-tetramethoxyflavone (**6**), 3, 5, 6, 7, 8, 3′, 4′-heptamethoxyflavone (**7**) and tangeretin (**8**)—the following natural coumarins: heraclenol (**1**), 8-geranyloxypsoralen (**2**), auraptene (**3**), and bergamottin (**4**). The utilization of preparative HPLC allowed their retrieval as pure compounds (purity >95% for each isolated molecule) in the form of amorphous colorless solids. Their retrieval yields from 1 kg of mandarin CPEO were depended on the fruit variety and the industrial procedure and determined as: heraclenol (**1**), 38–55 mg; 8-geranyloxypsoralen (**2**), 35–51 mg; auraptene (**3**), 22–33 mg; bergamottin (**4**), 14–19 mg, nobiletin (**5**), 4–11 mg; 5,6,7,4′-tetramethoxyflavone (**6**), 8,5–18 mg; 3,5,6,7,8,3′4′-heptamethoxyflavone (**7**), 51–103 mg; tangeretin (**8**), 39–80 mg. It must be noted that the retrieval yields of PMFs are comparable to those of the literature, while for natural coumarins this study constitutes the first literature report concerning their presence in mandarin CPEO [48].

With respect to the economic aspects of this study, because PMFs isolation has already been studied in detail previously [48], our interest was focused on the retrieval of natural coumarins. This endeavor presents a twofold significance, one concerning their applications and the second their market value. With respect to their potential applications and consecutive market interest and demand, it must be noted that the literature abounds with reports attributing diverse bioactivities to these natural compounds, indicating their significant value and numerous applications for the pharmaceutical industry and the emergence of developed methods for their efficient retrieval from agro-industrial byproducts. Specifically, the very rare furanocoumarin molecule heraclenol [51], found in species such as *Angelica* spp., *Citrus* spp., and *Heracleum* spp. [52], is known to display potent anti-inflammatory activity against TPA-induced ear edema [53]; it also acts as a potent activator for influencing platelet aggregation [52] and exhibits antiviral and in vitro inhibitory activities against HIV replication [54]. Recently, in terms of molecular docking exploitation, heraclenol was determined to act as a potent inhibitor of the main protease of SARS-CoV-2 virus (PDB ID: 5N5O) [55], highlighting a potential for future development against SARS-CoV-2. Finally, heraclenol exhibits significant antiproliferative properties against sensitive and resistant mouse T-lymphoma cells [56], which is indicative of its anti-cancer properties.

The molecule 8-geranyloxypsoralen, also known as xanthotoxol geranyl ether, is a linear furanocoumarin displaying potent antimicrobial activity against *Staphylococcus epidermidis*. This molecule also possesses intense antifungal properties against *Candida kruzei* and *Candida kefyr* [57] and nematocidal activity against *Bursaphelenchus xylophilus* [58]. Additionally, it acts as an inhibitor of cytochrome P450 3A4 (CYP3A4), an important enzyme of the liver that is important for xenobiotics oxidation [59]. In 2012, a study pointed out 8-geranyloxypsoralen as a potent inhibitor of β-secretase (BACE1) enzyme and displays high lipophilic potency in cells. Both features are crucial for the development of means for the prevention of Alzheimer’s disease appearance [60].

Auraptene possesses intense activities such as antidiabetic [61], antiprotozoal [62], anti-inflammatory [63], and immunomodulatory [64]. Additionally, auraptene displays inhibitory and chemo-preventive effects on the proliferation, tumorigenesis, and growth of several cancer cell lines by increasing the activity of glutathione S-transferase, formation of DNA adducts, and reduction in the number of aberrant crypt foci. Its anti-cancer properties are mediated by targeting different cell signaling pathways such as cytokines, genes modulating cellular proliferation, growth factors, transcription factors, and apoptosis [65]. One of the most important features of auraptene concerns its ability to prevent and/or treat different chronic diseases, such as cystic fibrosis and hypertension. Due to its intense anti-microbial and anti-inflammatory properties, the molecule auraptene acts as a potent therapeutic agent against periodontal diseases and for the prevention of *Porphyromonas gingivalis* adherence to oral epithelial cells [66]. In addition, auraptene exhibits multiple protective activities in the brain, displaying the ability to act as a neuroprotective for memory enhancement in rat models for Alzheimer’s disease [67].

Bergamottin exhibits various bioactivities, such as intense anticancer activity that is attributed to its anti-proliferation, anti-invasion, and anti-migration properties [68,69,70,71]. Moreover, literature reports indicate that this furanocoumarin displays the capability of inhibiting isoforms of cytochrome P450 (CYP) enzyme, particularly those of CYP3A4 and CYP1A1 [72]. The inhibitory activity of bergamottin for CYP1A1 is translated to an antimutagenic effect against the genotoxicity of this molecule in a *Salmonella typhimurium* microsome test [73]. Finally, a 2012 study concerning the antimycobacterium tuberculosis activity of *Citrus aurantifolia* hexane extract pointed out bergamottin as one of the most active constituents [74].

The market values of the retrieved natural compounds are considerably high, reflecting their bioactivities and respective demand by pharmaceutical industries. The lowest market prices identified on the internet (www.ChemFaces.com, accessed on 30 March 2021) for 1 mg of each natural compound are: EUR 54 for heraclenol, EUR 25 for 8-geranyloxypsoralen, EUR 20 for auraptene, and EUR 8 for bergamottin. Thus, in respect the obtained yields for each natural compound, it is estimated that the economic outcome of their retrieval from 1 kg of mandarin CPEO ranges from EUR 2052 to 2970 for heraclenol, EUR 875 to 1275 for 8-geranyloxypsoralen, EUR 440 to 660 for auraptene, and EUR 112 to 152 for bergamottin. Consequently, the overall financial result of these four compounds retrieval from each kg of mandarin CPEO ranges between EUR 3479 to 5057, revealing the economic importance and viability of the method, considering that the current market value of 1 kg of mandarin CPEO is between EUR 17 to 22 per 1 kg [43].

## 4. Materials and Methods

### 4.1. Mandarin CPEO

The CPEOs of mandarin used in this study were derived from the industrial juice-making process of *Citrus reticulata* Blanco and were kindly provided by the Christodoulou Bros S.A. fruit-juice making industry (Agia Triada–Nafplio, Greece). 

### 4.2. Solvents and Standards

Analytical grade isopropanol and methanol, which were used as solvents and HPLC grade water, methanol, and formic acid, used for the HPLC analyses, were obtained from Fisher (Waltham, MA, USA) Chemicals. Deuterated acetonitrile used for NMR spectroscopy was provided by Sigma-Aldrich ((St. Louis, MO, USA). 

All chemical standards used for the verification of natural products structures were purchased from Sigma-Aldrich (Darmstadt, Germany).

### 4.3. Retrieval of Natural Coumarins

#### 4.3.1. Isolation of Non-Volatiles Fraction

The non-volatile fraction of mandarin CPEO was separated through the exclusive evaporation of the volatile fraction. This was achieved under mild conditions (low temperature and reduced pressure), developed in terms of this endeavor. More specifically, 100 g of CPEO was placed into a Büchi Rotary Evaporator (model R-210, with a vacuum controller V-850, vacuum pump V-700, and heating bath B-491 (Flawil, Switzerland). and 100 mL of isopropanol was added. The alcohol forms an azeotropic mixture with D-limonene [75], the prevailing component of volatiles (>90%). Thus, a large proportion of volatiles is evaporated. Subsequently, additional amounts of isopropanol were added, and the evaporation was continued until the full removal of all volatiles and the separation of the non-volatiles fraction in the form of a slurry were achieved. The method was also applied on a pilot scale using a 25 L capacity rotary evaporator of COMECTA, Model COM-1020 (Barcelona, Spain) for the evaporation of the volatile fraction of 4 kg mandarin CPEO.

#### 4.3.2. Retrieval of Natural Coumarins Using Preparative HPLC

The non-volatile fraction was dissolved in methanol at a concentration of approximately 50 mg/mL and the mixture was introduced via manual injection into an HPLC system (Hewlett Packard series 1100 (Wilmington, NC, USA), with an injection volume of 200 μL and flow rate of 1.2 mL/min. A reverse-phase column Kromasil C18, 5 μm, 250 × 10 mm (MZ Analysentechnik) was used, with a respective guard column of the same material and company, all operated at room temperature. The mobile phase consisted of MeOH (solvent A) and H_2_O with 0.1% formic acid (solvent B) in accordance with the following gradient program: 0.0–5.0 min, A 60%, 5.0–25.0 min A 60%→80%, 25.0–30.0 min A 80%, 30.0–45.0 min A 80→90%, 45.0–50.0 min A 90%, 50.0–65.0 min A 90→100%, 65.0–75.0 min A 100%, 75.1–80.0 min A 60%. The outcome detection was achieved with a diode array detector of the same company (Hewlett Packard, series 1050, Wilmington, NC, USA) set at 280 nm. The retrieved natural coumarin and furanocoumarins were collected in respect to the peaks determined for the following retention times: heraclenol (**1**), 40.7 min; nobiletin (**5**), 50.2 min; 5, 6, 7, 4′-tetramethoxyflavone (**6**), 50.7 min; 3, 5, 6, 7, 8, 3′, 4′-heptamethoxyflavone (**7**), 51.9 min; tangeretin (**8**), 56.3 min; 8-geranyloxypsoralene (**2**), 68.1 min; auraptene (**3**), 72.9 min; bergamottin (**4**), 76.5 min. Then, the solvent of each fraction was evaporated under reduced pressure to afford the corresponding natural product as a colorless amorphous solid which was then stored in the freezer.

#### 4.3.3. Analytical HPLC

The above HPLC system was also used, in analytical mode, for the identification of natural compounds and the verification of their purity. Specifically, the respective samples obtained from preparative HPLC were injected into a Nucleosil 100-5 C18, 5 μm, 4.6 × 250 mm column obtained from Macherey-Nagel (Düren, Germany) and a guard column of the same material and company. The mobile phase consisted of methanol (solvent A) and H_2_O with 0.1% formic acid (solvent B), in accordance with the following gradient program: 0.0−17.5 min A 80→100%, 17.6−20.0 min A 80%. The injected volume was 20 μL, with a flow rate of 1.0 mL/min and a column temperature of 30 °C. The detector was set to monitor at two wavelengths, 280 and 254 nm.

#### 4.3.4. NMR Spectroscopy

Nuclear magnetic resonance (NMR) spectra (δ in ppm, *J* in Hz) were recorded on a Bruker Avance III 600 (600 MHz) NMR spectrometer (Bruker Biospin GmbH, Rheinstetten, Germany) using tetramethylsilane (TMS, Aldrich, St. Louis, MO, USA) as the internal reference δ = 0.00). The determination of chemical structures of the retrieved molecules was performed using data obtained from the following experiments: ^1^H–NMR, ^13^C–NMR, and the two-dimensional NMR experiments COSY (correlation spectroscopy), HMQC (heteronuclear multiple quantum coherence), and HMBC (heteronuclear multiple bond coherence).

## 5. Conclusions

The exploitation of mandarin CPEO, a scarcely investigated industrial byproduct, indicated its potential as a rich source of bioactive natural coumarins and furanocoumarins. The method developed herein refers to the evaporation, under mild conditions, of the volatile fraction and subsequent retrieval, using preparative HPLC, of the following natural coumarins and furanocoumarins in pure form: heraclenol, 8-geranyloxypsoralen, auraptene, and bergamottin. These molecules are known to possess significant bioactivities, displaying high demand by pharmaceutical companies. Thus, 1 kg of mandarin CPEO with a current market value ranging between EUR 17 to 22 is capable of producing high added value natural compounds worth EUR 3479 to 5057, indicating the great potential of this methodology for valorization in terms of the circular economy.

## Data Availability

Data is contained within the article.

## Sample Availability

Limited amounts of samples of the compounds are available from the authors.
